# Promotion of cardiac microtissue assembly within G-CSF-enriched collagen I-cardiogel hybrid hydrogel

**DOI:** 10.1093/rb/rbae072

**Published:** 2024-06-19

**Authors:** Hamid Khodayari, Saeed Khodayari, Malihe Rezaee, Siamak Rezaeiani, Mahmoud Alipour Choshali, Saiedeh Erfanian, Ahad Muhammadnejad, Fatemeh Nili, Yasaman Pourmehran, Reihaneh Pirjani, Sarah Rajabi, Naser Aghdami, Canan Nebigil-Désaubry, Kai Wang, Habibollah Mahmoodzadeh, Sara Pahlavan

**Affiliations:** Department of Stem Cells and Developmental Biology, Cell Science Research Center, Royan Institute for Stem Cell Biology and Technology, ACECR, Tehran 19395-4644, Iran; Department of Developmental Biology, University of Science and Culture, Tehran 13145-871, Iran; Cancer Research Center, Cancer Institute of Iran, Tehran University of Medical Sciences, Tehran 1419733141, Iran; Department of Stem Cells and Developmental Biology, Cell Science Research Center, Royan Institute for Stem Cell Biology and Technology, ACECR, Tehran 19395-4644, Iran; Department of Stem Cells and Developmental Biology, Cell Science Research Center, Royan Institute for Stem Cell Biology and Technology, ACECR, Tehran 19395-4644, Iran; Department of Stem Cells and Developmental Biology, Cell Science Research Center, Royan Institute for Stem Cell Biology and Technology, ACECR, Tehran 19395-4644, Iran; Department of Stem Cells and Developmental Biology, Cell Science Research Center, Royan Institute for Stem Cell Biology and Technology, ACECR, Tehran 19395-4644, Iran; Department of Cell Engineering, Cell Science Research Center, Royan Institute for Stem Cell Biology and Technology, ACECR, Tehran 19395-4644, Iran; Cancer Biology Research Center, Cancer Institute of Iran, Tehran University of Medical Sciences, Tehran 1419733141, Iran; Department of Pathology, Cancer Institute, Imam Khomeini Hospital Complex, Tehran University of Medical Sciences, Tehran 1419733141, Iran; Department of Stem Cells and Developmental Biology, Cell Science Research Center, Royan Institute for Stem Cell Biology and Technology, ACECR, Tehran 19395-4644, Iran; Department of Developmental Biology, University of Science and Culture, Tehran 13145-871, Iran; Obstetrics and Gynecology Department, Arash Women’s Hospital, Tehran University of Medical Sciences, Tehran 1653915981, Iran; Department of Stem Cells and Developmental Biology, Cell Science Research Center, Royan Institute for Stem Cell Biology and Technology, ACECR, Tehran 19395-4644, Iran; Department of Cell Engineering, Cell Science Research Center, Royan Institute for Stem Cell Biology and Technology, ACECR, Tehran 19395-4644, Iran; Department of Regenerative Medicine, Cell Science Research Center, Royan Institute for Stem Cell Biology and Technology, Tehran 19395-4644, Iran; Institute National de le santé et de la recherce médicale, INSERM, University of Strasbourg, UMR 1260-Regenerative Nanomedicine, CRBS, Central of Research in biomedicine of Strasbourg, Strasbourg 90032, France; Department of Physiology and Pathophysiology, School of Basic Medical Sciences, State Key Laboratory of Vascular Homeostasis and Remodeling, Peking University, Beijing 100191, China; Cancer Research Center, Cancer Institute of Iran, Tehran University of Medical Sciences, Tehran 1419733141, Iran; Department of Stem Cells and Developmental Biology, Cell Science Research Center, Royan Institute for Stem Cell Biology and Technology, ACECR, Tehran 19395-4644, Iran

**Keywords:** hydrogel, cardiac tissue engineering, human pluripotent stem cell, G-CSF, angiogenesis

## Abstract

Tissue engineering as an interdisciplinary field of biomedical sciences has raised many hopes in the treatment of cardiovascular diseases as well as development of *in vitro* three-dimensional (3D) cardiac models. This study aimed to engineer a cardiac microtissue using a natural hybrid hydrogel enriched by granulocyte colony-stimulating factor (G-CSF), a bone marrow-derived growth factor. Cardiac ECM hydrogel (Cardiogel: CG) was mixed with collagen type I (ColI) to form the hybrid hydrogel, which was tested for mechanical and biological properties. Three cell types (cardiac progenitor cells, endothelial cells and cardiac fibroblasts) were co-cultured in the G-CSF-enriched hybrid hydrogel to form a 3D microtissue. ColI markedly improved the mechanical properties of CG in the hybrid form with a ratio of 1:1. The hybrid hydrogel demonstrated acceptable biocompatibility and improved retention of encapsulated human foreskin fibroblasts. Co-culture of three cell types in G-CSF enriched hybrid hydrogel, resulted in a faster 3D structure shaping and a well-cellularized microtissue with higher angiogenesis compared to growth factor-free hybrid hydrogel (control). Immunostaining confirmed the presence of CD31^+^ tube-like structures as well as vimentin^+^ cardiac fibroblasts and cTNT^+^ human pluripotent stem cells-derived cardiomyocytes. Bioinformatics analysis of signaling pathways related to the G-CSF receptor in cardiovascular lineage cells, identified target molecules. The *in silico*-identified STAT3, as one of the major molecules involved in G-CSF signaling of cardiac tissue, was upregulated in G-CSF compared to control. The G-CSF-enriched hybrid hydrogel could be a promising candidate for cardiac tissue engineering, as it facilitates tissue formation and angiogenesis.

## Graphical abstract

**Figure:**
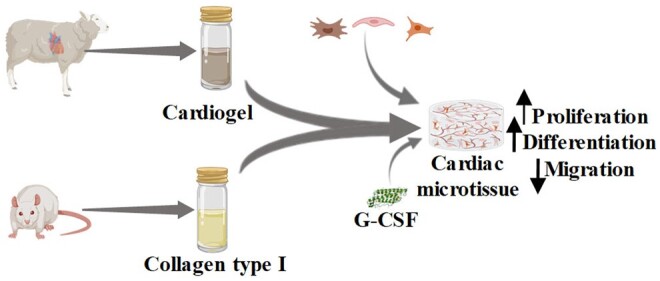

## Introduction

Cardiovascular diseases (CVDs) are a major cause of global mortality and morbidity, affecting the quality of life for patients [1]. Importantly, CVDs might occur secondary to other organs’ disorders such as diabetes, or result from drug toxicity particularly chemotherapeutics-related cardiotoxicities [2]. Consequently, there is an undeniable necessity for in-depth investigations of CVDs with respect to the underlying mechanisms and pathophysiology, as well as drug-induced cardiotoxicity, for a more accurate and prompter diagnostic and prognostic approach along with prevention of drug toxicities. To this end, recent *in vitro* platforms for mechanistic studies revealed an unprecedented promise toward this goal [3].

In the past few decades, tissue engineering has emerged as a focal point for research and development, encompassing both *in vitro* modeling and therapeutic options [4]. The capacity to fabricate engineered cardiac models presents a novel perspective for comprehending diverse facets of the physiology and pathophysiology of cardiac disorders [5, 6]. These fabricated cardiac microtissues enable precise exploration of cellular processes and functions within a controlled microenvironment, mimicking human disease biology more closely compared to animal models [5, 6]. Notably, numerous studies have been implemented to assess potential drug toxicity in cardiac microtissue and to facilitate drug screening [7]. Additionally, engineered cardiac microtissues have been employed to delve into rare cardiac disorders, including genetic heart conditions [8].

While conventional two-dimensional (2D) culture systems proved effective in studying cardiac function using both primary culture as well as human pluripotent stem cells-derived cardiovascular lineage cells, these systems exhibit inherent limitations such as lack of three-dimensional (3D) cell–cell and cell–extracellular matrix interactions [9–11]. To address these challenges, tissue engineering technology emphasizes the integration of biomaterials as substrates alongside cardiac lineage cells, thus creating an environment more suitable to conduct precise *in vitro* cardiovascular research [9, 12]. Natural and synthetic biomaterials are employed to establish scaffolds, forming a 3D platform for generating heart microtissues [12]. Notably, hydrogels as a subset of 3D hydrophilic polymer networks, have gained substantial prominence within cardiac tissue engineering. Their capacity to enhance cell adhesion, to facilitate the deposition of extracellular matrix (ECM) and to support cellular growth, underpins their significance [13, 14].

In the context of cardiac microtissue generation, cardiac decellularized ECMs (cdECM) emerge as a frequently employed biomaterial due to the biochemical composition and reservoir of growth factors. The replication of native microenvironment and the promotion of normal physiological behavior in cardiac lineage cells, render cdECM as a preferred choice among natural biomaterials for cardiac tissue engineering [15–17]. cdECM-derived hydrogels exhibit noteworthy physical and chemical properties, creating an environment akin to the *in vivo* milieu and facilitating localized delivery of growth factors [18–20]. However, the application of certain hybrid ECM/hydrogels, such as collagen I-containing ones, has also demonstrated enhanced properties in terms of physical characteristics and stem cell maturation [21]. Despite the advancements in cardiac tissue engineering and the creation of *in vitro* cardiac microtissue models, the challenge of vascularization remains fundamental in the field of biomedical sciences. The absence of mature and functional vasculature significantly restricts the size and complexity of these models [22]. In the present study, a hybrid hydrogel comprising collagen type I (ColI) and cdECM was fabricated, in order to obtain a substrate for cardiac tissue engineering with appropriate physical properties. The Granulocyte colony-stimulating factor (G-CSF)-enrichment of hybrid hydrogel improved cardiovascular lineage cell migration toward tissue formation. Furthermore, the cellularity and angiogenesis of the engineered cardiac microtissue was well enhanced along with higher incidence of cTNT^+^ cells derived from differentiation of human pluripotent stem cells-derived cardiac progenitor cells (hPSC-CPCs). STAT3 was identified as one of the majors signaling molecules for G-CSF function in cardiac microtissue formation.

## Materials and methods

### Hydrogel preparation and characterization

#### Decellularization of heart tissue and evaluations

##### Heart tissue decellularization

Sheep hearts were purchased from slaughterhouse and transferred to the laboratory in a cold box. Each heart was then trimmed off excess fat, valves and ligament tissues. The remaining cardiac tissue was cut into several pieces and frozen for 24 h at −20°C. Thereafter, the pieces were subjected to a decellularization process using a method described previously [23]. Briefly, the heart pieces were washed with double distilled water (DDW) for 5 h followed by 48 h of treatment with a 1% (w/v) solution of sodium dodecyl sulfate (SDS, Sigma L4390). Subsequently, the pieces were immersed in a 1% (w/v) solution of Triton X-100 for 30 min. The resulting pieces were rinsed with DDW, while being gently stirred for 24 h. Finally, the decellularized cardiac tissue was lyophilized, ground to a fine powder, and stored at 4°C.

##### Histological assessment

The intact and decellularized cardiac tissues were fixed in a 10% formalin solution (pH 7.4) at 25°C for 24 h. Afterward, the samples were rinsed with DDW and dehydrated through a serial ethanol dilution before being embedded in paraffin. The resulting paraffin-embedded tissues were sliced into sections that were 5 μm thick, rehydrated and subjected to staining using hematoxylin and eosin (H&E, Sigma-Aldrich), Masson’s trichrome (Tr.M, Sigma-Aldrich), Alcian blue (Alc.B, Sigma-Aldrich) and 4′,6-diamidino-2-phenylindole (DAPI, Sigma-Aldrich, D8417). The resulting samples were imaged using a microscope (Olympus, BX51).

##### DNA quantification

The samples were homogenized and dissolved in a lysis buffer containing tris-HCl (50 mM), EDTA (50 mM), SDS (1%) and NaCl (10 mM) with a pH adjusted to 8.0. The samples were then incubated with proteinase k in a water bath at 65°C for a period of overnight digestion. A phenol-chloroform extraction was performed, followed by DNA precipitation using 100% ethanol and washing with 70% ethanol. Finally, the resulting extracts were immersed in RNase-free water overnight, and the DNA concentrations were measured at 280 nm using a nanodrop spectrophotometer (Thermo Scientific™, NanoDrop One/One^C^). The average DNA content was determined based on three independent replicates and expressed as μg/mg of dry sample weight. The isolated DNA sample of native and decellularized cardiac muscle was also qualitatively assessed using the 2% agarose gel electrophoresis and subjected to imaging with a gel documentation system.

##### Immunohistofluorescence staining

The intact and decellularized tissue sections were subjected to an antigen retrieval step and subsequently permeabilized in a solution containing PBS and 0.5% Triton X-100 at room temperature (RT) for 20 min. Afterward, the slides were blocked in a solution of PBS and 10% heat-inactivated bovine serum albumin (BSA) at RT for 45 min. Thereafter, the slides were incubated overnight with primary antibodies, including rabbit polyclonal anti-fibronectin (Abcam, ab2413), anti-laminin (Abcam, ab66155) and anti-collagen I (Abcam, ab21286). Finally, the samples were incubated with a florescent conjugated goat anti-rabbit secondary antibody (TermoFisher, A11037) and subjected to nuclear staining with DAPI. The immunostained samples were imaged using an Olympus IX71 fluorescence microscope equipped with an Olympus DP72 digital camera.

#### Extraction and characterization of collagen type I

##### Collagen type I extraction from rat tail

All animal experiments were performed under the guidance and in conformity with protocols approved by the Institutional Ethics Committee at Royan Institute (IR.ACECR.ROYAN.REC.1401.113). Type I collagen was extracted from rat tail tendons using a protocol described previously [24]. In Brief, after amputation of the rat tails, the tendons were dissected. The samples were subjected to a process involving acetic acid solution to generate a sterile soluble collagen product. Initially, the samples were washed in PBS for 20 min with stirring, followed by immersing in acetone 99% for 5 min to remove fat. After washing of samples, they were immersed in a solution containing 0.02 M of acetic acid under agitation at 4°C for 24 h. Following, the resulting solution containing collagen underwent centrifuge at 10 000 rpm and 4°C for 30 min. Subsequently, the crude collagen was frozen at a temperature of −20°C. Following freezing, the collagen was then lyophilized to produce a sponge-like material that could be stored at a temperature of −20°C.

##### SDS PAGE

To check the quality of collagen extraction, SDS PAGE was performed. Initially, a 10% resolving gel and a 4% stacking gel composed of acrylamide 40%, Tris 1.5 M (PH: 8.8), DDW, SDS 10%, ammonium persulfate 10% and tetramethyl ethylenediamine, were prepared. A solution, with a concentration of 2.5 mg/ml extracted collagen in 0.05 M acetic acid, 1 mg/ml of standard collagen (Sircol collagen assay kit, Biocolor) and a standard ladder was subjected to SDS PAGE using a Mini Dual Vertical Electrophoresis Unit. Approximately 11 µl of the prepared solutions were loaded into each well. The resulting gels were stained using a solution of 0.1% Coomassie brilliant blue R250 dissolved in a mixture of water, methanol and acetic acid (in a volumetric ratio of 5:4:1, respectively), and subsequently detained using a solution composed of methanol, distilled water and acetic acid (in a volumetric ratio of 5:4:1, respectively).

#### Preparation of hybrid hydrogel and evaluations

##### Hydrogel formation from decellularized heart tissue

The Cardiac ECM hydrogel (Cardiogel: CG) was prepared according to a previously published protocol with some modifications [25]. The powder of cdECM underwent sterilization with UV for 20 min. Subsequently, 2 mg/ml of the powder was digested in a solution of 0.1 M HCl and 1 mg/ml pepsin (Merck, 107185) for about 48 h under constant stirring at low temperature. For gelation, the pH of the digested hydrogel was neutralized by adding a cold solution of sodium hydroxide (NaOH) in 10× PBS (1M). The gelation of CG was evaluated in different concentrations of NaOH by incubation at 37°C for 30 min.

##### Hybrid hydrogel formation from CG and collagen type I

To prepare collagen hydrogel, the shredded pieces of collagen sponge were sterilized under UV for 20 min, followed by dilution in a 0.05-M solution of acetic acid with a concentration of 5 mg/ml under constant stirring at a temperature of 4°C for 24 h. The ColI hydrogel was prepared by neutralizing the solution with a cold 1 M NaOH.

To prepare hybrid hydrogel, each hydrogel solution was initially made; the CG with a concentration of 1 mg/ml and ColI with a concentration of 2.5 mg/ml in cold DMEM/F12. Afterward, the hybrid hydrogel (ColI-CG) was prepared in two ratios of 1:1 and 1:2 by mixing CG and ColI. The gelation of ColI-CG hybrid hydrogel was evaluated by incubation at 37°C for 30 min.

##### Compression assay

To assess the mechanical properties of hydrogels, a compression assay was performed using HCT-25/400 Zwick/Roell system. For this purpose, 1 mg/ml CG, 2.5 mg/ml ColI and 1:1 and 1:2 ratios of ColI-CG were prepared, and 300 µl of each hydrogel with 8 mm diameter and 14 mm height after gelation, was placed on the lower surface of the system. Subsequently, the upper surface of the system loaded a linear pressure with a speed of 1 mm/s on hydrogels. The parameters of stress (KPa) and strain (%) were calculated using the parameters of extension (mm) and force (N), which were obtained from the system, based on the following formulas:

σ=F/A (σ:stress, F:force, A:area)

$$\varepsilon =\Delta L/L_{0}\left(\varepsilon :\text{strain},\;\Delta L:\text{change of length},\;L_{0}:\text{initial length}\right)$$

### Cell culture and characterization

#### Expansion of primary fibroblasts

The human foreskin fibroblasts (hFFs) and human cardiac fibroblasts (hCFs) were received from Royan Stem Cell Bank (Royan Institute, Tehran). The hFFs and hCFs were expanded in the culture medium composed of DMEM/F12 (Gibco, 31331028) supplemented with 15% fetal bovine serum (FBS), 1% nonessential amino-acids (NEAA; Gibco, 11140-035), 1% penicillin/streptomycin (Gibco, 15070-063) and 1% GlutaMAX (Thermo Scientific, 35050038) at 37°C and 5% CO_2_. Cells were passaged when the confluency reached 80%. The hCFs were characterized by immunostaining for vimentin.

#### Expansion of human embryonic stem cells-derived cardiac progenitor cells

The human embryonic stem cells-derived cardiac progenitor cells (hESC-CPCs) were received as a gift from Prof. Hossein Baharvand’s lab (Royan Institute, Tehran) [26]. The cells were seeded onto the Matrigel-coated plates and expanded in a medium composed of DMEM/F12 supplemented with 2% B-27 without vitamin A (Gibco, 12587-010), 2 mM L-glutamine, 1% NEAA, 0.1 mM β-mercaptoethanol and freshly added chosen factors, 0.5 µM A83-01 (Stemgent, 04-0014), 100 ng/ml bFGF (Sigma-Aldrich) and 3 µM CHIR99021 (Stegment). The culture medium was renewed every other day and the passage of cells were done when the confluency reached 80%. The hESC-CPCs were characterized by immunostaining for CPC specific marker; NKX2.5.

#### Expansion of human umbilical vein endothelial cells

The human umbilical vein endothelial cells (HUVECs) were received from Royan Stem Cell Bank (Royan Institute, Tehran). The cell expansion was performed in EGM-2 medium (Sigma-Aldrich, C-39221) containing 20% FBS, 1% NEAA, 1% penicillin/streptomycin, 1% GlutaMAX at 37°C and 5% CO_2_. The culture media was renewed every other day. The passage of cells was done when the confluency reached 80%. The HUVECs were characterized by immunostaining for CD31.

#### Immunocytofluorescence staining

For immunostaining, the hCFs, hESC-CPCs, and HUVECs were cultured onto the 4-well plates in the previously explained media. After 80% confluency, the cells were subjected to fixation in a 4% paraformaldehyde solution at 4°C for 20 min, permeabilization and blocking in PBS/1% BSA/0.1% Triton X-100 at RT for 45 min, incubation with primary antibodies [mouse polyclonal anti-Vimentin antibody (Santa Cruz Biotechnology, SC6260), rabbit polyclonal anti-NKX2.5 antibody (Abcam, ab189202) and rabbit polyclonal anti-CD31 antibody (Abcam, ab28364)] at 4°C overnight, and incubation with secondary antibodies [goat anti-rabbit antibody (TermoFisher, A11037) and donkey anti-mouse antibody (Abcam, ab150105)] at RT for 1 h. The cells were finally counterstained with DAPI (2 µg/ml) at RT for 2 min in order to mark nuclei. The immunostained cells were visualized using a fluorescent microscope (Olympus, IX71, Japan).

### Biocompatibility assessment

In order to assess the biocompatibility of hybrid hydrogel, LIVE/DEAD assay was performed on ColI-CG-encapsulated hFFs. Initially, 1.5 × 10^6^ hFFs/ml was added to the ColI-CG pre-gel and slowly resuspended. Thereafter, 100 µl of hFFs/pre-gel suspension was added into each well of a 48-well culture plate and incubated at 37°C for 30 min in order to form a gel. Subsequently, the hybrid hydrogel-encapsulated hFFs were cultured at 37°C and 5% CO_2_ and the media were exchanged every other day. To assess the viability of encapsulated cells at 1-, 3- and 7-days post-culture, 150 μl of LIVE/DEAD staining solution (containing PBS with 3 μM calcein AM and 2.5 μM PI) was applied to each sample and incubated at 37°C for 30 min. The cells were visualized under a fluorescence microscope (Olympus, IX71), with viable cells staining green and dead cells staining red. The number of green and red cells was counted offline in 3 field of views for each sample, using the ImageJ software (Fiji using Java 6). In addition to hybrid hydrogel, the biocompatibility of each single hydrogel (CG and ColI) was assessed, too.

### Preparation of G-CSF enriched hybrid hydrogel, cardiac microtissue formation and characterization

#### Cardiac microtissue fabrication using G-CSF enriched hybrid hydrogel

The ColI-CG pre-gel at a ratio of 1:1 was mixed with 1000 ng/ml of recombinant G-CSF (Filgrastim, AryaTinaGene). Subsequently, the G-CSF-enriched ColI-CG was used as an extracellular matrix in co-culture of hESC-CPCs, HUVECs and hCFs at a ratio of 5:3:2 and final cell density of 1.5 × 10^6^. 100 µl of pre-gel/cell suspension was added into each well of a 48-well culture plate and incubated at 37°C for 30 min to form a gel. The ColI-CG without G-CSF was used as control in the experiments of microtissue formation. The microtissues were cultured in a media composed of EGM-2 and CPC media at a ratio of 1:1 at 37°C and 5% CO_2_. The culture medium was replaced every other day and the microtissues were harvested at Day 7. The formation of 3D microtissue structures was monitored over time and imaged for hybrid hydrogel contraction analysis. Furthermore, the viability of hydrogel-encapsulated cells was evaluated using the LIVE/DEAD staining at Day 7.

#### Histological assessments

The microtissues were fixed, processed and subjected to H&E, Tr. M and Alc.B staining at Days 3 and 7 of culture according to the protocol described in 1.1.2. The stained sections were imaged using BX51 Olympus microscope.

#### Immunohistochemistry staining

To check the abundance and distribution of each cell type, the microtissue sections were subjected to immunohistochemistry staining using cTNT, CD31 and Vimentin antibodies. Briefly, the microtissue sections that were prepared by PFA fixation, paraffin embedding and microtome slicing as described in 1.1.2, were stained with primary antibodies (goat polyclonal anti-cTNT antibody, rabbit polyclonal anti-CD31 antibody and mouse polyclonal anti-Vimentin antibody) at 4°C overnight. Horseradish peroxidase-conjugated secondary antibody (Chromogen System-HRP (DAB), Dako, k3468) was then incubated with tissue sections for immunohistochemistry analysis. Samples were counterstained with hematoxylin and images were taken using an Olympus microscope (Olympus, BX51) equipped with an Olympus DP72 digital camera. In each image, 10 fields of view were analyzed with respect to the total number of cells (based on nuclear staining) and the percentage of positive cells as well as the intensity of the reaction product and, given scores 0–8 (Allred Score). Scores 0 and 2 were considered negative, while scores 3–8 considered positive.

#### Gene expression analysis

Total RNA was isolated from cardiac microtissues using the TRIzol reagent (Ambion) following the guidelines provided by the manufacturer. The quality of the extracted RNA was evaluated by gel electrophoresis. cDNA synthesis was carried out using the TaKaRa PrimeScript 1st strand cDNA synthesis kits, followed by Real-time qRT-PCR performed on a Rotor-Gene™ 6000 Real-Time PCR System (Corbett Life Science). The samples were collected from three independent biological replicates. The expression level of the genes was normalized to glyceraldehyde 3-phosphate dehydrogenase (*GAPDH*) as a reference gene. The analysis was performed using the comparative CT method (2-ΔΔct). A list of the primers used for real-time qRT-PCR are presented in Table S1.

### *In silico* analysis and experimental evaluation of G-CSF signaling pathways

#### Bioinformatics analysis

In order to investigate the signaling pathways related to the G-CSF receptor (G-CSF-R) in cardiovascular lineage cells, the downstream genes of G-CSF-R was first obtained from GeneCard database (https://pathcards.genecards.org/card/signaling_by_csf3_(g-csf)). Afterward, the keywords of ‘Adult Heart’, ‘Fetal Heart’, ‘Fetal_HCA_Heart’, and ‘TS_Heart’ were searched in the list of the downstream genes using Web-based Cell-type Specific Enrichment Analysis of Genes (WebCSEA) (see https://bioinfo.uth.edu/webc) database. The results were analyzed and visualized by using GGPLOT2 package of R-studio software. Furthermore, the genes involved in the maturation of human fetal cardiomyocytes were extracted from MSigDB database (msigdb.org/gsea/msigdb-https://www.gsea) for the study ‘GOBP_REGULATION_OF_CARDIOBLAST_DIFFERENTIATION.v20 23.1. Hs’ with code M24044. Subsequently, the protein–protein interaction (PPI) between the downstream genes of G-CSF-R and genes involved in the maturation of human fetal cardiomyocytes was obtained using STRING database and analyzed by CytoScape software (version 3.8.0).

#### Gene expression profiling

The expression of several cardiac-related genes with the highest interaction with downstream genes of G-CSF-R, was evaluated in the cardiac microtissues using real-time qRT-PCR. These genes included *STAT-3*, *Connexin43 (Cx43)*, *HIF1-α*, *VEGF*, *c-MYC*, *KI67*, *MMP-2* and *CXCR-4*. The list of primers is presented in Table S2.

#### Immunohistochemistry analysis

To check VEGF, MMP-2 and KI67 expression in cardiac microtissue sections, immunohistochemistry was conducted on Day 7 samples. After the initial preparations as described in ‘Immunohistochemistry staining’, the samples were incubated overnight at 4°C with primary antibodies targeting VEGF (Abcam, ab2350), MMP2 (Abcam, ab97779) and KI67 (Abcam, ab16667). Following treatment with secondary antibody at 37°C for 1 h, the samples were also treated with a horseradish peroxidase-conjugated secondary antibody (Chromogen System-HRP (DAB), Dako, k3468) to visualize the stained proteins by immunohistochemistry. Finally, the samples were counterstained with hematoxylin and visualized using an Olympus microscope (Olympus, BX51) equipped with an Olympus DP72 digital camera, and analyzed using Allred Scoring system. For Ki67, 10 fields of view were analyzed in each image with respect to the total number of cells (based on nuclear staining) and number of antibody-stained cells. Then, the percentage of positive cells were calculated by the following formula:

(Number of antibody−stained cells/Total number of calls)*100.

### Statistical analysis

Data are presented as mean ± standard deviation (SD). Statistical analysis was performed using GraphPad-Prism software (GraphPad Prism 8.4.3) and Student *t*-test was utilized for comparison between two groups, while one-way analysis of variance (ANOVA) was employed for comparison between more than two groups. *P* < 0.05 was considered statistically significant.

## Results

### Decellularization did not alter ECM proteins

**Figure 1.**
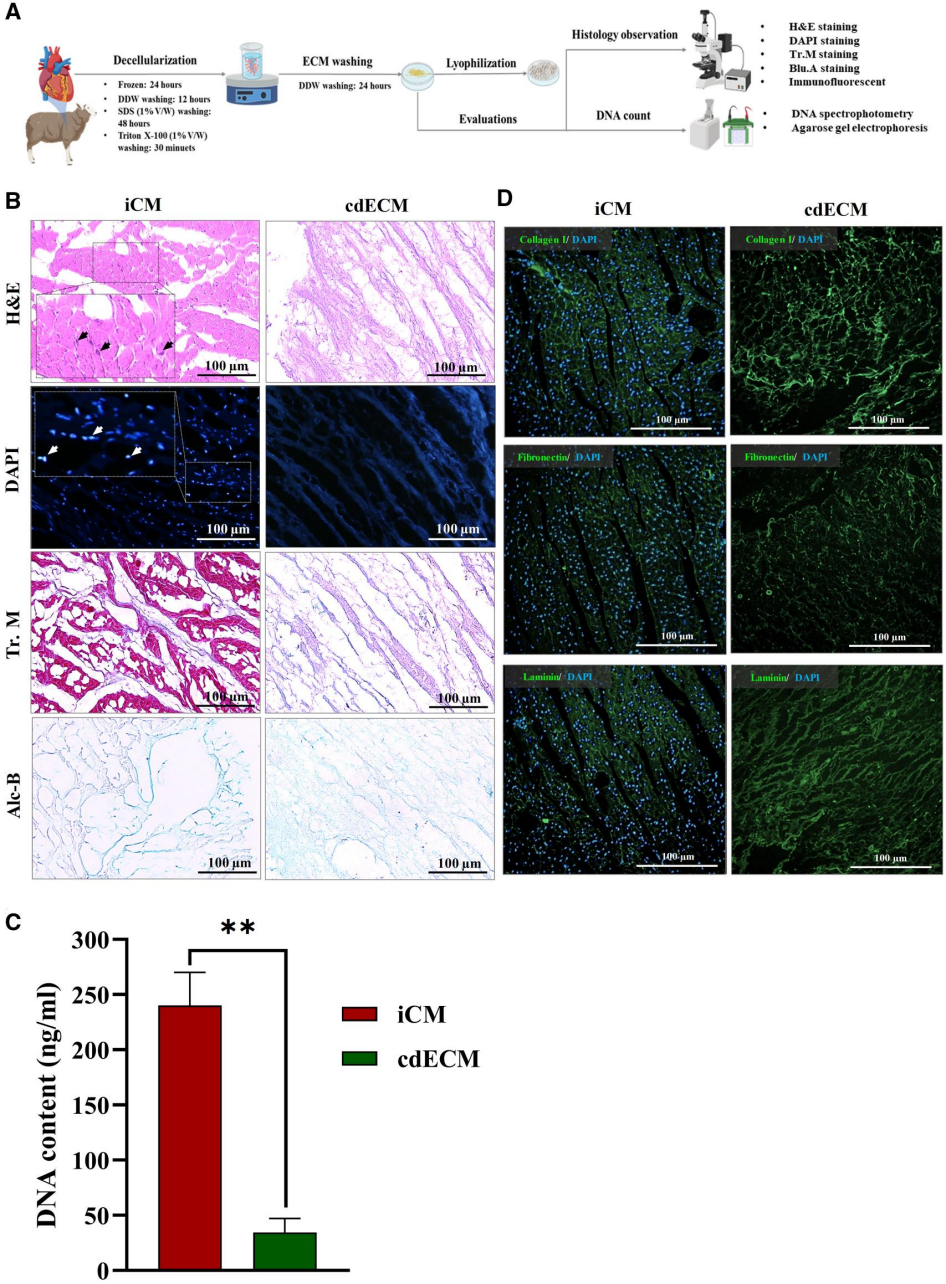
Characterization of heart decellularization. (**A**) Schematic representation of sheep heart decellularization and evaluations. (**B**) Histological assessments validate the complete removal of cells and the preservation of the ECM structure following decellularization. The arrows are pointing cell nuclei in the iCM samples. (**C**) DNA quantification of cdECM reveals effective DNA elimination, showing a significant difference from the iCM (*n* = 3, **P < 0.01). (**D**) Immunohistofluorescence staining for collagen I, fibronectin and laminin confirms the preservation of these ECM components in cdECM samples. iCM, intact cardiac muscle; cdECM, cardiac decellularized extracellular matrix; H&E, hematoxylin and eosin; Tr.M, Masson’s trichrome; Alc.B, Alcian blue; DAPI, 4′,6-diamidino-2-phenylindole.

In order to examine the efficacy of the decellularization protocol, decellularized samples of sheep cardiac tissues were histologically analyzed and subjected to DNA content evaluation (Figure 1A). H&E staining results clearly demonstrated the absence of cell nuclei in the cdECM, with the ECM remaining intact post-decellularization (Figure 1B). DAPI staining further supported the successful decellularization, as evidenced by the lack of stained cell nuclei in the cdECM compared to the intact cardiac muscle (iCM) (Figure 1B). Histological findings from Tr.M and Blu.A staining indicated the preservation of collagen and glycosaminoglycan after the decellularization process (Figure 1B). In comparison to the iCM, a substantial reduction of DNA was observed in the cdECM, confirming the effective removal of cells during decellularization (Figure 1C and Figure S1). Approximately, 85% of the DNA content was removed in the cdECM (34 ng/ml) in contrast to the iCM group (240 ng/ml) (Figure 1C). Furthermore, immunohistofluorescence staining of ECM proteins (collagen I, fibronectin and laminin), revealed their robust preservation in all cdECM samples (Figure 1D).

### Collagen type I integration improved the stiffness of CG

**Figure 2.**
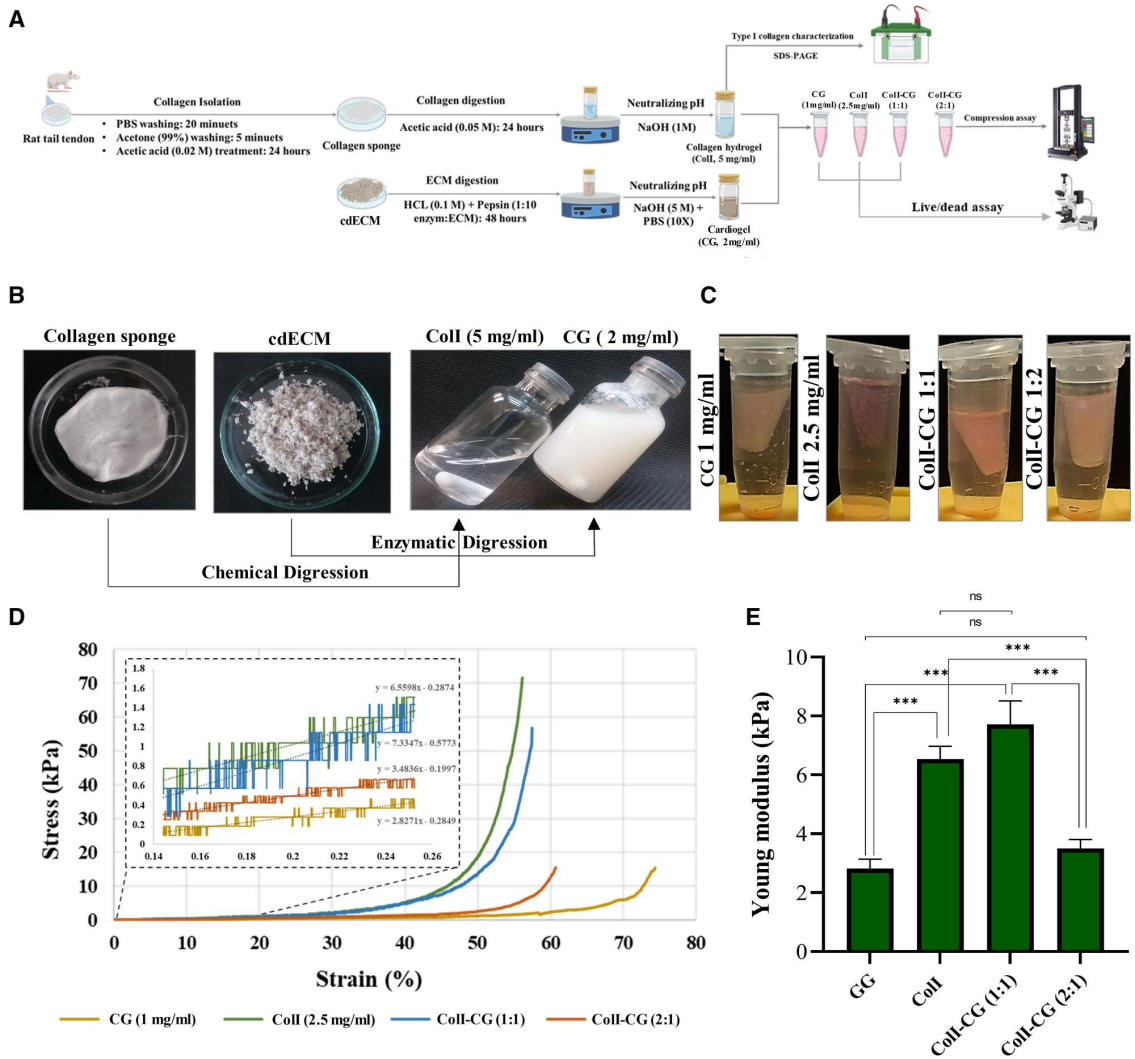
Creation of the hybrid hydrogel and compression analysis. (**A**) A schematic representation of the hybrid hydrogel preparation and evaluations. (**B**) Freeze-dried collagen sponge and cdECM chemical and enzymatic digestion separately to form the hydrogel. (**C**) The gelation status of CG and ColI hydrogels individually, as well as two hybrid ColI-CG hydrogels in 1:1 and 1:2 ratios, following a 30-min incubation at 37°C, immersed in PBS buffer. (**D**) Compression stress–strain curves for CG hydrogel, ColI hydrogel and the two hybrid ColI-CG hydrogels. The stiffness of each hydrogel is indicated at a 50% threshold of strain. (**E**) Young’s modulus quantification results (*n* = 3, ***P < 0.001). ECM, extracellular matrix; cdECM, cardiac decellularized extracellular matrix; ColI, collagen type I; CG, cardiogel; ColI-CG, collagen I cardiogel hybrid hydrogel; ns, not significant.

Fabrication of hydrogels for cardiac tissue engineering should involve the optimization of mechanical properties and conductivity. We have previously fabricated a polypyrrole-incorporated cardiogel (CG-PPy), which showed superior electrical conductivity compared to CG alone [25]. However, the mechanical property of CG-PPy could only be improved by using cross-linking [25]. To keep the composition of the fabricated hydrogel as natural as possible and to avoid chemical reactions for enhancement of mechanical properties, we assessed the impact of ColI integration on cardiogel (CG) stiffness. To do so, rat tail collagen was isolated and the successful production of the hydrogels from the cdECM and collagen sponge was achieved using the established protocols (Figure 2A and B). The SDS PAGE results confirmed the presence of three ColI subunits (α1, α2 and β) in the ColI at the concentration of 2.5 mg/ml (Figure S2). CG and ColI, at concentrations of 1 mg/ml and 2.5 mg/ml, respectively, as well as two hybrid ColI-CG hydrogels at 1:1 and 1:2 ratios, exhibited successful gelation at pH 7.4 following a 30 min incubation at 37°C (Figure 2C). We assessed the mechanical properties of the CG alone and following integration with ColI using a compression assay.

At a 50% threshold of strain, a significant increase in the gel stiffness of ColI-CG hybrid hydrogel was observed at the ratio of 1:1 (14 kPa) compared to CG (1.7 kPa) (Figure 2D). However, the ColI-CG hybrid hydrogel at 2:1 ratio displayed a reduction in its stiffness (2 kPa) (Figure 2D). In this experiment, the ColI exhibited the highest stiffness (19.5 kPa), where its integration to CG at 1:1 ratio reinforced the mechanical property of ECM hydrogel (Figure 2D). These findings were well reflected in Young’s modulus, where ColI and ColI-CG hybrid hydrogel (1:1) showed substantially greater values (6.54 ± 0.43 and 7.71 ± 0.8, respectively) compared to CG (Figure 2E).

### Collagen I integration improved biocompatibility and cell retention of CG

**Figure 3.**
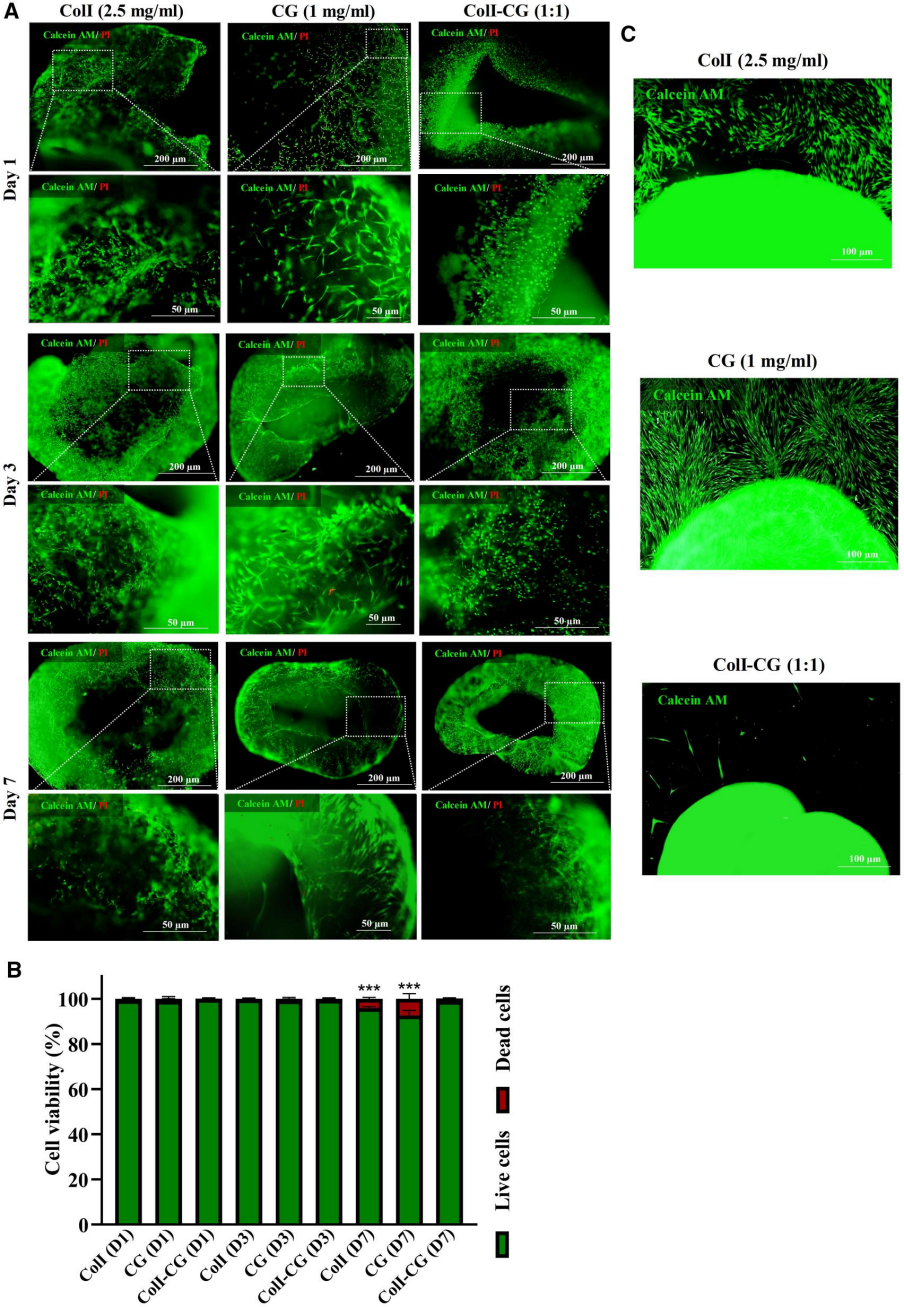
*In vitro* experiments on survival and trans-hydrogel migration of encapsulated hFFs. (**A**) Fluorescence microscopy images capture hFFs encapsulated within ColI (2.5 mg/ml), CG (1 mg/ml) and ColI-CG 1:1 hybrid hydrogel at 1-, 3- and 7-days post-culture, stained with calcein AM and PI. (**B**) Quantified results from the LIVE/DEAD assay provide a clear representation of the percentage of cell viability (*n* = 3, ***P < 0.001), highlighting significant differences among the groups. (**C**) Further fluorescence microscopy images reveal trans-hydrogel migrated cells from ColI, CG and ColI-CG 1:1 hybrid hydrogel after 7 days of culture, with green cells indicating the presence of migrated cells within the culture dish substrate. ColI, collagen type I; CG, cardiogel; ColI-CG, collagen I cardiogel hybrid hydrogel; PI, propidium iodide.

As the hybrid hydrogel is considered to accommodate the various cell types of cardiac tissue, it should have no cytotoxic effect and provide a biocompatible microenvironment. Therefore, the hFFs as a readily available cell source were cultured in ColI-CG 1:1 hybrid hydrogel as well as ColI (2.5 mg/ml) and CG (1 mg/ml) as control groups. The cells were subjected to LIVE/DEAD staining at three distinct time points of Day 1, Day 3 and Day 7, where the percentages of live cells consistently exceeded 99% across all three experimental groups on Days 1 and 3 (Figure 3A and B). However, a pivotal turning point emerged on Day 7 that unveiled a compelling insight. On this day, both the ‘ColI’ and ‘CG’ groups exhibited a significant increase in the percentage of dead cells (4.14% and 7.35%, respectively) compared to the ColI-CG (1.2%) (Figure 3B). Notably, the fluorescent images of Calcein AM-stained cells revealed a relevant decrease in the number of trans-hydrogel migrated cells in the ColI-CG compared to the ColI and CG alone, after 7 days of culture (Figure 3C). These findings underscore the potential of the ColI-CG hybrid hydrogel to enhance not only the viability, but also the retention of hydrogel-encapsulated cells, presenting a promising platform for cardiac tissue engineering in practice.

### G-CSF-enriched hybrid hydrogel improved cardiac microtissue formation by promoting migration and angiogenesis

**Figure 4.**
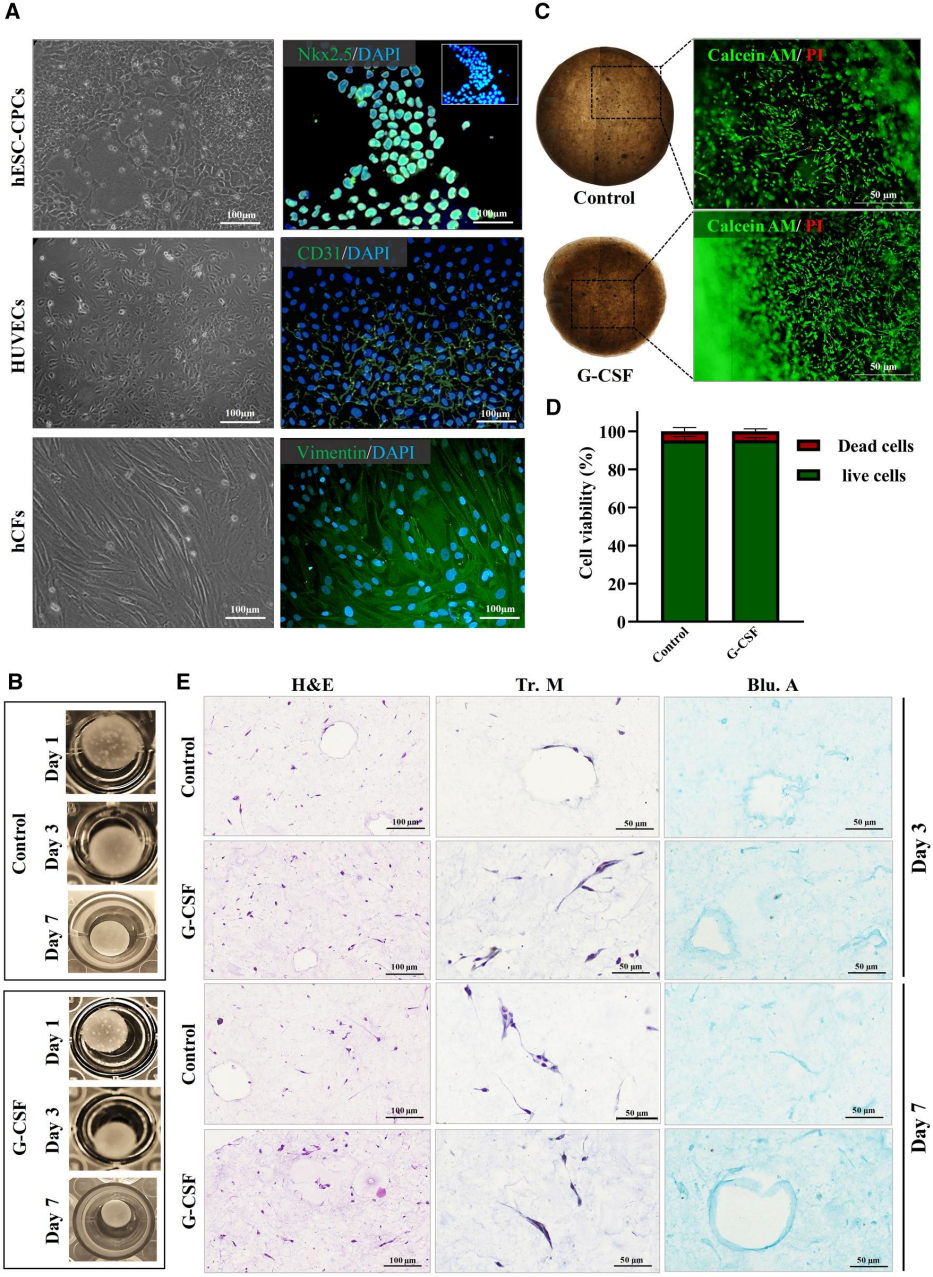
Cardiac microtissue fabrication using G-CSF enriched hybrid hydrogel. (**A**) Representative light and fluorescence microscopy images of hCFs, hESC-CP Cs and HUVECs. Cytoplasmic marker (vimentin), nucleus marker (NKX2.5) and membrane marker (CD31) were respectively utilized for immunofluorescence staining and characterization of hCFs, hESC-CP Cs and HUVECs. Cell nuclei were stained with DAPI. (**B**) Macroscopic images showcase the trend of cardiac microtissues formation from control and G-CSF samples at 1-, 3- and 7-days post-culture. (**C**, **D**) Microscopic images and corresponding quantified results from the LIVE/DEAD assay demonstrate no significant differences in the number of dead cells after 7 days of microtissue culture in both control and G-CSF samples (*n* = 3). (**E**) Histological assessments are presented for evaluating cellularity, capillary formation and ECM remodeling in control and GCSF cardiac microtissues at 3- and 7-days post coculture. hCFs, human cardiac fibroblasts; hESC-CP Cs, human embryonic stem cell-derived cardiac progenitor cells; HUVECs, human umbilical vein endothelial cells; PI, propidium iodide; ns, not significant; H&E, hematoxylin and eosin; Tr.M, Masson’s trichrome; Alc.B, alcian blue; DAPI, 4′,6-diamidino-2-phenylindole.

*In vitro* cardiac microtissue formation facilitated the studies of cardiac physiology and pathology as well as the assessment of cardiotoxicity [5, 6]. To fabricate engineered tissues using ColI-CG hybrid hydrogel, various cell types of heart tissue were used (Figure S3). hCFs, hESC-CPCs and HUVECs were cultured, expanded and characterized for their specific markers (Figure 4A). NKX2.5^+^ hESC-CPCs, CD31^+^ HUVECs and vimentin^+^ hCFs were cocultured in ColI-CG at a density of 1.5 × 10^6^ cells/ml and the ratio of 5:3:2 (Figure S3). To explore the impact of G-CSF on cardiac microtissue formation, the same composition of the cells was cocultured in the G-CSF enriched hybrid hydrogel. While both cells containing CG-ColI (Control) and 1000 ng/ml G-CSF enriched hybrid hydrogel (G-CSF), started to form 3D structures after 24 h, the latter exhibited a higher contraction rate as evidenced in lower diameters of fabricated microtissues at Days 1, 3 and 7 (Figure 4B). Both hydrogels supported cell viability similarly after 7 days (Figure 4C and D). H&E staining revealed an even distribution of cells throughout both hydrogels at Days 3 and 7 (Figure 4E), with G-CSF showing substantially higher number of cells in investigated HPFs compared to Control (57.7 ± 3.2 vs. 37.33 ± 2.5 at Day 3 and 74.7 ± 4.7 vs. 51.7 ± 3.1 at Day 7). Furthermore, the presence of capillary-like structures was evident in both Control and G-CSF groups at Days 3 and 7 of coculture. Collagen bundles were more frequent around cell populations of G-CSF group compared to Control as identified by Tr.M staining (Figure 4E). In line with collagen composition, the glycosaminoglycan content of G-CSF group appeared to be higher compared to Control (Figure 4E). These findings revealed a positive impact of G-GSF on ECM remodeling of engineered microtissue.

**Figure 5.**
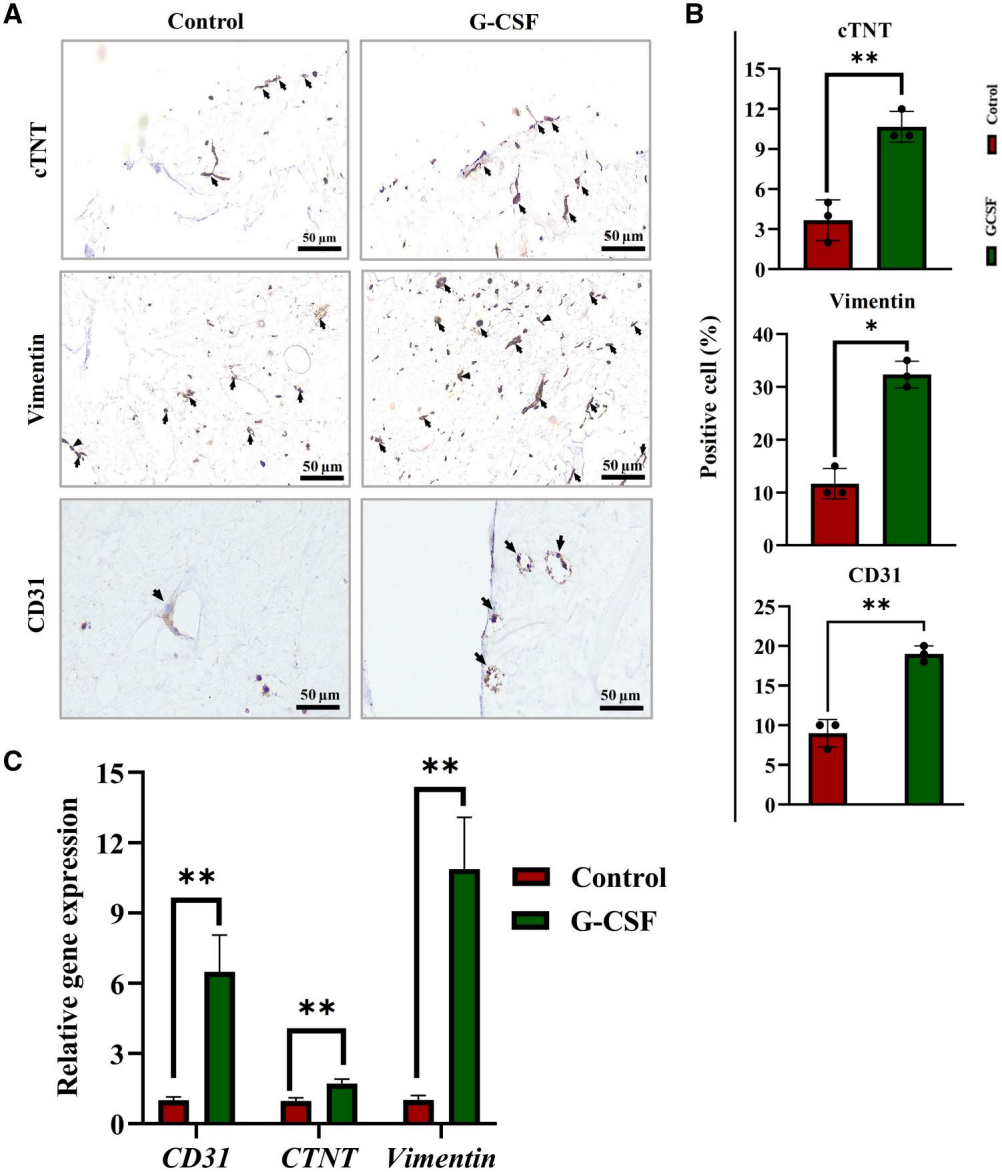
Immunohistochemical and gene expression analysis of CTNT, vimentin and CD31 in cardiac microtissue samples. (**A**) Representative IHC images of cardiac microtissues in control and G-CSF groups. Cytoplasmic markers CTNT and vimentin and membrane marker CD31 were employed for IHC staining to identify and trace cardiomyocytes, fibroblasts and endothelial cells, respectively. The cell nuclei were counterstained with hematoxylin. (**B**) Quantitative results of the IHC staining results using the Allred scoring system, where no expression is denoted as 0, weak expression as 1, moderate expression as 2 and strong expression as 3 (*n* = 3, *P < 0.05). (**C**) Relative gene expression analysis of endothelial marker CD31, cardiomyocyte marker CTNT and fibroblast marker vimentin in cardiac microtissues through qRT-P CR analysis (*n* = 3, *P < 0.05 and **P < 0.01). CTNT, cardiac troponin T; IHC, immunohistochemistry.

When the microtissues were evaluated with respect to cell types, a significant increase in the cTNT^+^ cardiomyocytes, vimentin^+^ hCFs and CD31^+^ endothelial cells were observed in G-CSF compared to Control (Figure 5A and B). The results of protein staining were well correlated with gene expression results (Figure 5C), which highlights the positive impact of G-CSF enrichment on differentiation (higher cTNT^+^ cardiomyocytes derived from hPSC-CPCs) and cell retention (greater vimentin^+^ and CD31^+^ cells). Furthermore, the G-CSF group contained a substantially higher number of CD31^+^ vessel-like structures (Figure 5A), which indicated a proangiogenic condition in this group.

### STAT3 functions downstream of G-CSF in cardiac microtissue formation

**Figure 6.**
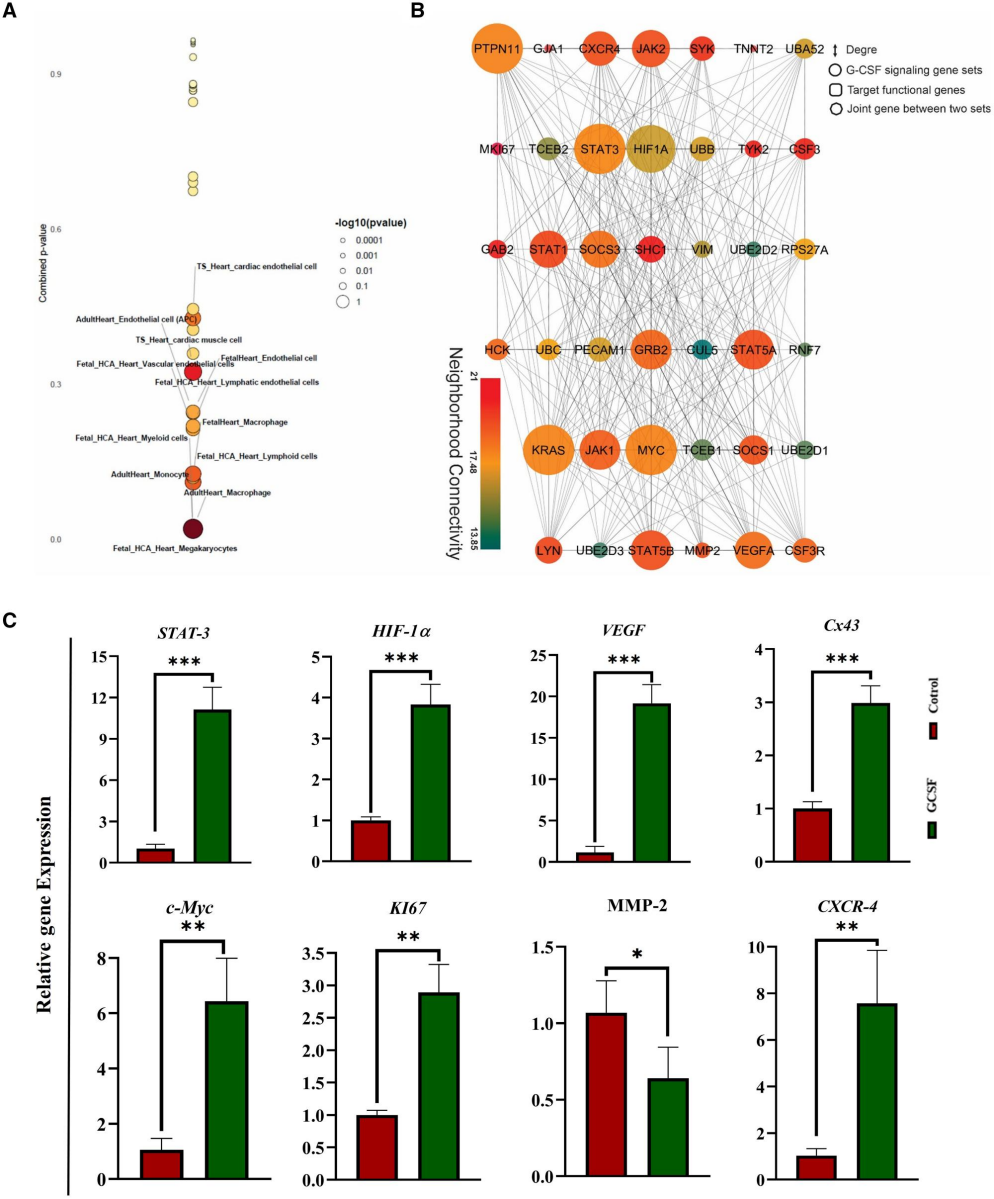
Bioinformatics analysis and gene expression evaluation of G-CSF-related pathways in cardiac microtissue samples. (**A**) Bubble plot diagram illustrates the results of cell enrichment analysis for downstream genes of the G-CSF receptor signaling pathway across different cardiac lineages. Bubbles represent different cell types, with their size and color indicating their ranking (**B**) PPI diagram depicts the protein–protein interactions within the G-CSF signaling pathway and its relationship to cardiovascular differentiation. Circular nodes represent proteins associated with G-CSF signaling, square nodes represent proteins involved in differentiation and octagonal nodes denote proteins common to both G-CSF signaling and cardiovascular differentiation. Node size reflects the degree of interactions, while node color signifies their interactions with neighboring proteins. (**C**) Relative gene expression analysis of candidate genes (*STAT-3*, *Connexin43*, *HIF1-α*, *VEGF*, *c-MYC*, *KI67*, *MMP-2* and *CXCR-4*) in cardiac microtissues using qRT-PCR assays (*n* = 3, *P < 0.05, **P < 0.01, and ***P < 0.001). PPI, protein–protein interaction.

The results of cell-type enrichment analysis obtained from the WebCSEA system, showed that the genes involved in the G-CSF signaling pathway are significantly correlated with various cell types of cardiovascular lineage (*P* < 0.05). The Bobble plot in Figure 6A presents the identified cell lineages which are ranked from 0 to 1 based on the *P* values, where the size and color of the bubbles mark the rank of the cell type. Accordingly, monocytes, myeloid cells and megakaryocytes of cardiac tissue had the highest correlation, with the endothelial cells gaining next ranks.

The PPI diagram shows the interaction of G-CSF signaling pathway proteins with those involved in cardiovascular lineage cell differentiation (Figure 6B). Circular nodes represent proteins involved in G-CSF signaling, square nodes for proteins of differentiation and the octagonal nodes show the common proteins between G-CSF signaling and cardiovascular differentiation. The size of the nodes indicates the degree or number of interactions and the color stands for interaction with their neighboring proteins. Accordingly, PTPN11, STAT-3 and KRAS rank first with 26 interactions among all proteins active in the G-CSF signaling pathway. Furthermore, the results showed that other STAT family proteins including STAT-5a/b and STAT-1 reside in higher ranks with 22 and 21 interactions, respectively (Figure 6B). On the other hand, higher ranks belonged to MYC (26 interactions), HIF-1α (24 interactions), VEGFA (21 interactions) and CXCR-4 (20 interactions) in the cardiovascular differentiation proteins. Notably, although MMP-2 (nine interactions) and MKI67 (eight interactions) did not rank high with respect to the number of interactions, but mainly interacted with active proteins of PPI network. Based on this *in silico* analysis, G-CSF signaling is mainly active in immune cells of cardiac tissue as well as endothelial cells and functions through STAT-3 (Figure 6B).

**Figure 7.**
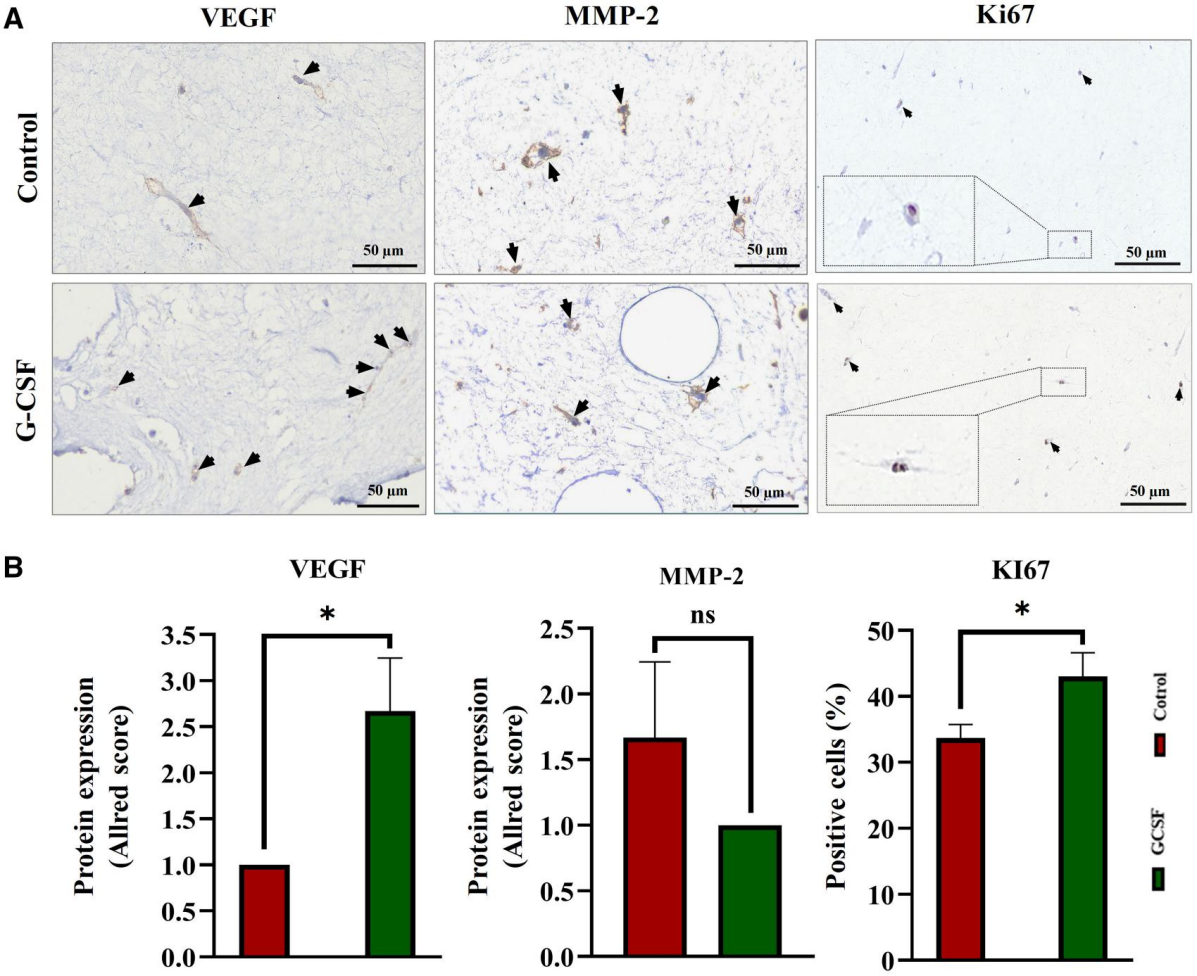
Immunohistochemical and gene expression analysis of VEGF, MMP-2 and KI67 in cardiac microtissue samples. (**A**) Representative IHC images of cardiac microtissues in control and G-CSF groups. Targeted proteins VEGF, MMP-2 and KI67 were employed for IHC staining to identify the angiogenesis, cell migration and proliferation, respectively. The cell nuclei were counterstained with hematoxylin. (**B**) Quantitative results of the IHC staining results using the Allred scoring system, where no expression is denoted as 0, weak expression as 1, moderate expression as 2 and strong expression as 3 (*n* = 3, *P < 0.05). IHC, immunohistochemistry; VEGF, vascular endothelial growth factor; MMP-2, matrix metalloproteinase-2.

Interestingly, the gene expression analysis confirmed the upregulation of *STAT-3*, *HIF-1a*, *VEGF*, *c-MYC* and *KI67*, *Cx43* and *CXCR4* in the G-CSF group compared to control (Figure 6C). However, *MMP-2* was downregulated in G-CSF-enriched microtissues (Figure 6C). While VEGF^*+*^ and Ki67^+^ cells were present with significantly higher frequencies in G-CSF group compared to control, MMP-2^+^ cells did not differ between the two microtissue types (Figure 7A and B). These findings further unraveled the mechanistic aspects of the G-CSF impact on the promotion of proliferation, migration, differentiation and angiogenesis in the process of cardiac microtissue formation.

## Discussion

Recently, there has been a growing interest in developing engineered cardiac tissue that effectively mimics the microenvironment of cardiomyocytes within the heart. This involves using materials that have similar physicochemical characteristics to the native ECM of the heart, as well as being biocompatible and relevant to natural cardiac tissue [27]. In this regard, natural biomaterials, specially cdECM-derived hydrogels, have been found to be more effective in providing the aforementioned properties, compared to synthetic materials [28, 29]. So far, cdECM-derived scaffolds have been widely used in fabricating *in vitro* cardiac microtissues for studying cardiotoxicity and modeling cardiac disorders, which can provide valuable tools for mechanistic studies including transcriptome, proteome and signaling pathways involved [30, 31]. Consequently, it is not surprising that decellularized hearts could serve as attractive options for cardiac tissue engineering [16, 31, 32]. However, one significant challenge that remains is the formation of a vascular network and angiogenesis in these engineered tissues. Therefore, biomaterials with angiogenic properties similar to native tissue could be the more practical alternatives [22, 33]. Herein, we assessed the physicochemical properties and angiogenic potential of a G-CSF enriched hybrid hydrogel derived from collagen I integration into cardiogel. This innovative hydrogel demonstrated remarkable results in terms of cell proliferation, differentiation as well as angiogenesis, contributing to an improved microtissue formation. In addition, the bioinformatic analysis suggested that the pro-angiogenic effects of G-CSF could be mediated by STAT3.

Depletion of tissue cellular contents plays a crucial role in the generation of cdECM-based scaffolds. It is widely recommended that cdECM should contain less than 50 ng of dsDNA per mg of ECM dry weight after undergoing decellularization procedure [34]. Besides, it is essential to effectively preserve the ECM components and architecture by using appropriate decellularization protocol and enzymatic treatment [35]. In this line, a diverse range of decellularization techniques have been explored in order to decellularize cardiac tissue, from which physical approaches including freeze-thaw, detergent-based approaches including SDS and Triton X100, or combination of physical and detergent-based approaches are mostly used [36]. Additionally, pepsin-mediated ECM digestion followed by pH neutralization and incubation at 37°C, is considered as the most frequent gelation method for cdECM used in the experimental studies [18]. Moreover, multiple studies demonstrated that scaffolds derived from cdECM could provide a similar microenvironment to native heart tissue with respect to biomechanical and biochemical properties, leading to proper support during the proliferation and differentiation of cardiac lineage cells [37–39]. Accordingly, a previous study demonstrated that a combination of freeze-thaw and treatment with detergents, including 0.5% SDS and 1% Triton X-100, successfully achieved the decellularization of cardiac tissue (dsDNA: 34.26 ng/ml) [40]. This approach effectively preserved important ECM components such as ColI, laminin and fibronectin. Moreover, cardiogel, prepared using a pepsin-mediated protocol, exhibited no cytotoxic effects on hESC-derived CPCs and HUVECs. Further analysis revealed significant overexpression of *c-TNT* and *α-MHC*, as cardiac-specific genes, as well as the presence of c-TNT positive cardiomyocytes in the microtissue fabricated from cardiogel and hESC-CPCs, suggesting its capability to support cardiac lineage cell differentiation [40].

In the current study, we aimed to improve the mechanical properties of cardiogel by collagen I integration and attribute angiogenic potential by G-CSF enrichment. The physical properties of a natural hydrogel used for 3D experiments, are believed to have a notable impact on all aspects of tissue formation, especially for a contractile cardiac tissue [41]. The composition of hydrogels play a determining role in physical stiffness and is one the most important issues in hydrogel fabrication to construct an engineered tissue [42–44]. Among the ECM elements, ColI showed a more determining function in the physical integrity of myocardial ECM [45], as well as cardiomyocyte development [33, 46] and activities, particularly electrophysiological activity [47]. In this regard, a study demonstrated that addition of 3 mg rat tail collagen to Matrigel-based hydrogel could improve the mechanical stiffness of the hydrogel. This study also revealed that combined hydrogel with ColI facilitated the ESC-derived cardiomyocytes maturation, by upregulation of MLC2v, a maturation marker of ventricular myocytes, as well as TNNI3, TNNI2, MYH6/7, as sarcomeric proteins. It was suggested that these effects probably depend on the stiffness of the hydrogel [21]. Consistently, our results demonstrated that addition of 2.5 mg/ml ColI to CG at ratio of 1:1, significantly improved stiffness of the hydrogel. Furthermore, the viability and attachment of fibroblasts were significantly improved in this hybrid hydrogel, compared to CG or ColI hydrogel alone. As a result, we used this hybrid hydrogel to construct cardiac microtissue by incorporating the cardiovascular lineage cells, including CPCs, HUVECs and CFs, which showed promising results. Similar to this study, previous investigation used the co-culture of cardiac lineage cells into agarose to create cardiac microtissue [48].

Angiogenesis is one the most vital components in the construction of the cardiac microtissues that aim to precisely mimic the native myocardium. So far, various approaches were developed to induce angiogenesis in engineered tissues, with a particular focus on the utilization of pro-angiogenic growth factors [22]. In this line, the significant promotion of angiogenesis was observed in the PDGF-enriched injectable nanofibers [49] and the IGF-1/HGF-enriched injectable alginate scaffold [50]. In the current study, we attempted to enrich the hybrid hydrogel with recombinant G-CSF, as an angiogenic factor, to induce angiogenesis in the cardiac microtissue. Likely, G-CSF-loaded gelatin-based hydrogel has demonstrated an increase in CD31^+^ and α-SMA^+^ cells, indicating promotion of the capillary density and vessel formation [51]. Mechanistically, G-CSF was found to increase the expression of SDF-1α, a potent cell homing agent [52]. The potential angiogenic effects of chemical immobilization of SDF-1α into cdECM-derived scaffolds have been shown [53]. We observed that loading of G-CSF accelerated the process of microtissue formation and increased its collagen density, which could imply on effect of G-CSF on ECM production and remodeling of microtissue. Also, G-CSF-enriched hydrogel demonstrated a notable increase in cell proliferation. The increased CD31^+^ cell population and vessel-like structures could indicate the role of G-CSF in inducing angiogenesis. In addition, significant upregulation of *Vimentin, cTNT* and *CD31* expression (as specific markers of cardiac fibroblast, cardiomyocyte and endothelial cell, respectively) suggested that G-CSF could improve the ability of the hydrogel in supporting the cells retention as well as differentiation of CPCs into cardiomyocytes. A previous study showed that G-CSF-enriched scaffolds could provide a promising microenvironment for enhancing cell proliferation and differentiation toward cardiac lineage. Also, G-CSF loading was associated with overexpression of connexin 43, cell elongation, and emergence of cellular junctions that closely resemble the typical arrangement of cardiomyocytes [54].

The binding of G-CSF to its receptor activates several signaling pathways, including Jak, STAT family and Erk signaling involved in endothelial cell proliferation, migration and adhesion, resulting in angiogenesis induction [55, 56]. The bioinformatic analysis illustrated that G-CSF receptor mostly interacted with STAT3, which was highly associated with VEGF, HIF-1α, KI67, c-MYC, MMP-2 and CXCR-4 proteins. Our experimental data indicated a significant increase in the expression of *STAT3* gene in hydrogels with G-CSF loading, compared to those without G-CSF. In addition, the expression of angiogenic factors (*VEGF* and *HIF-1α*) increased, indicating the angiogenic potential of G-CSF. However, cell migration-related factor (*MMP-2*) was downregulated in Day 7 microtissues, which may suggest an increased adhesion tendency of cells to ECM. It is worth noting that migration was enhanced at the initial stages of microtissue formation in the G-CSF-enriched hybrid hydrogel, as evidenced by faster 3D structure shaping. However, we did not check the expression of *MMP-2* at various time points, which should be taken into account. Increased expression of *KI67* and *c-MYC* genes indicated the effects of G-CSF-mediated STAT3 on cell proliferation.

In conclusion, our study supports the potential of a hybrid hydrogel derived from cardiogel and ColI for cardiac tissue engineering. This hydrogel effectively supports the survival and differentiation of CPCs into cardiomyocytes and has the potential for *in vitro* fabrication of vascularized cardiac microtissues, particularly when used in conjunction with G-CSF. Based on our bioinformatics and experimental analysis, G-CSF can induce angiogenesis within the microtissues by targeting STAT3.

## Supplementary Material

rbae072_Supplementary_Data

## Data Availability

The datasets used and/or analyzed during the current study are available from the corresponding author on reasonable request.
