# Supplementary material for: Promotion of cardiac microtissue assembly within G-CSF-enriched collagen I-cardiogel hybrid hydrogel
Source: Regen Biomater. 2024 Jun 19;11:rbae072. doi: 10.1093/rb/rbae072 (PMC11226883; doi:10.1093/rb/rbae072)
Supplement: rbae072_Supplementary_Data [file rbae072_supplementary_data.docx]

**Supporting Information**

| Supplementary Table 1. List of the primers used for qRT-PCR are represented. | |
| --- | --- |
| Gene | Primer sequences 5'---► 3' |
| *GAPDH* | F: AGGGTCTCTCTCTTCCTCTTGTGCTCT  R: CCAGGTGGTCTCCTCTGACTTCAACAG |
| *CD31* | F: AGCAGTACCACTTCTGAACTCC  R: AGGAATTGCTGTGTTCTGTGG |
| *cTNT* | F: ATGATGCATTTTGGGGGTTA  R: CAGCACCTTCCTCCTCTCAG |
| *Vimentin* | F: AAACTTAGGGGCGCTCTTGT  R: TGAGGGCTCCTAGCGGTTTA |

| Supplementary Table 2. List of the primers used for qRT-PCR are represented. | |
| --- | --- |
| Gene | Sequences 5'---► 3' |
| *GAPDH* | F: AGGGTCTCTCTCTTCCTCTTGTGCTCT  R: CCAGGTGGTCTCCTCTGACTTCAACAG |
| *KI67* | F: GATCGTTCCTTCAGGTATG  R: TCTTGAGCTTTCTCATCAGG |
| *STAT3* | F: GAAGAATCCAACAACGGCAG  R: TCACAATCAGGGAAGCATCAC |
| *CXCR-4* | F: AACTTCAGTTTGTTGGCTGC  R: CATTTCCTCGGTGTAGTTATCTG |
| *VRGF* | F: CTACCTCCACCATGCCCAGT  R: CCTCGGCTTGTCACATTTTT |
| *VEGF-R* | F: AAGTAATCCCAGATGACAACCA  R: GTTTGCACTCCAATCTCTATCAG |
| *HIF-1a* | F: CACCACAGGACAGTACAGGAT  R: CGTGCTGAATAATACCACTCACA |
| *c-MYC* | S:5' GCG TCC TGG GAA GGG AGA TCC GGA GC 3'  As: 5' TTG AGG GGC ATC GTC GCG GGA GGC TG 3' |
| *MMP2* | F: ATTGTATTTGATGGCATCGCTC  R: ATTCATTCCCTGCAAAGAACAC |
| *Cx43* | F: 5' AGG TGG ACT GTT TCC TCT CTC 3'  R: 5' TTG CTC ACT TGC TTG CTT GTT G 3' |

**
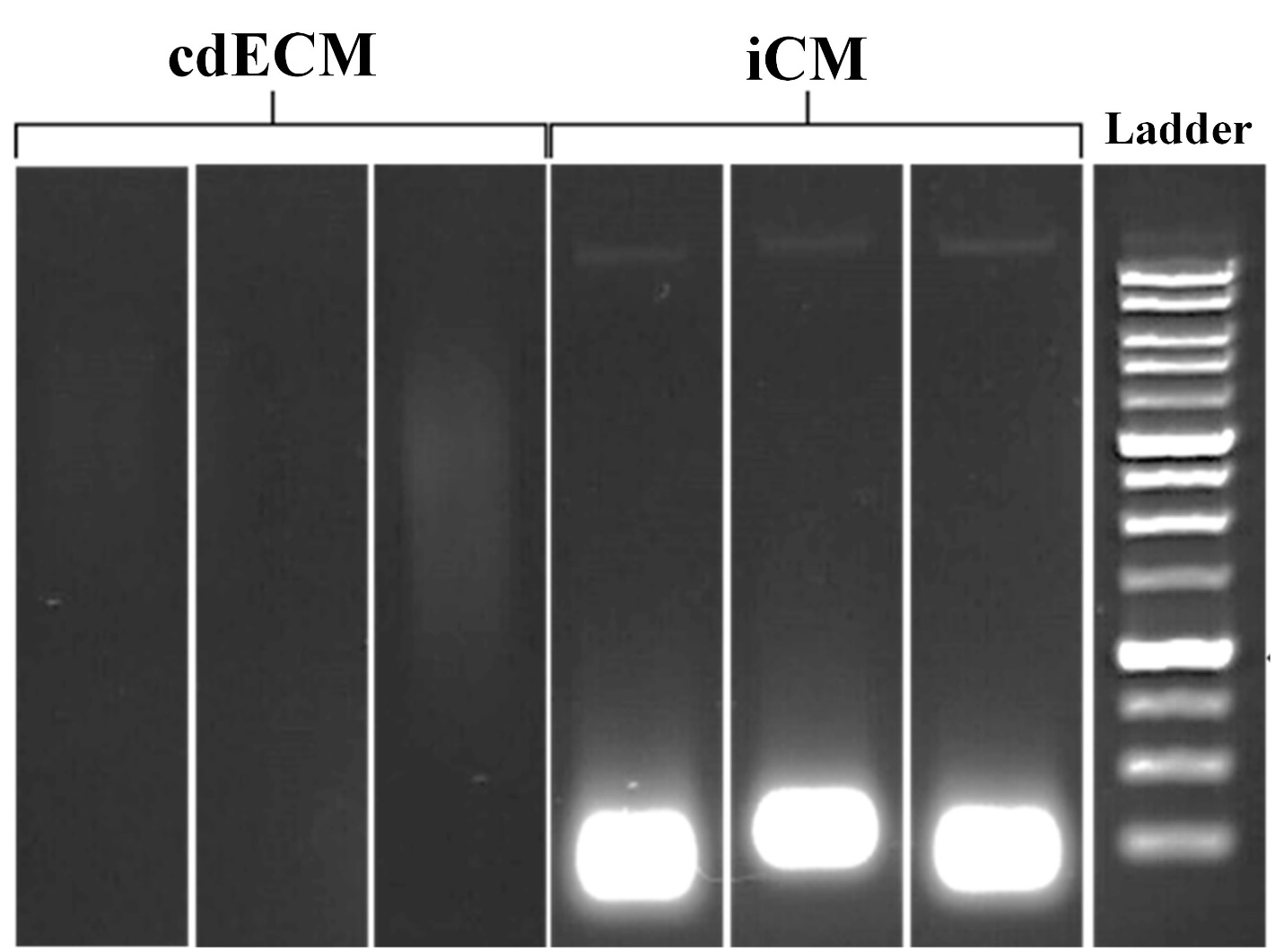
**

**Figure S1.** Agarose gel electrophoresis of extracted DNA.

**
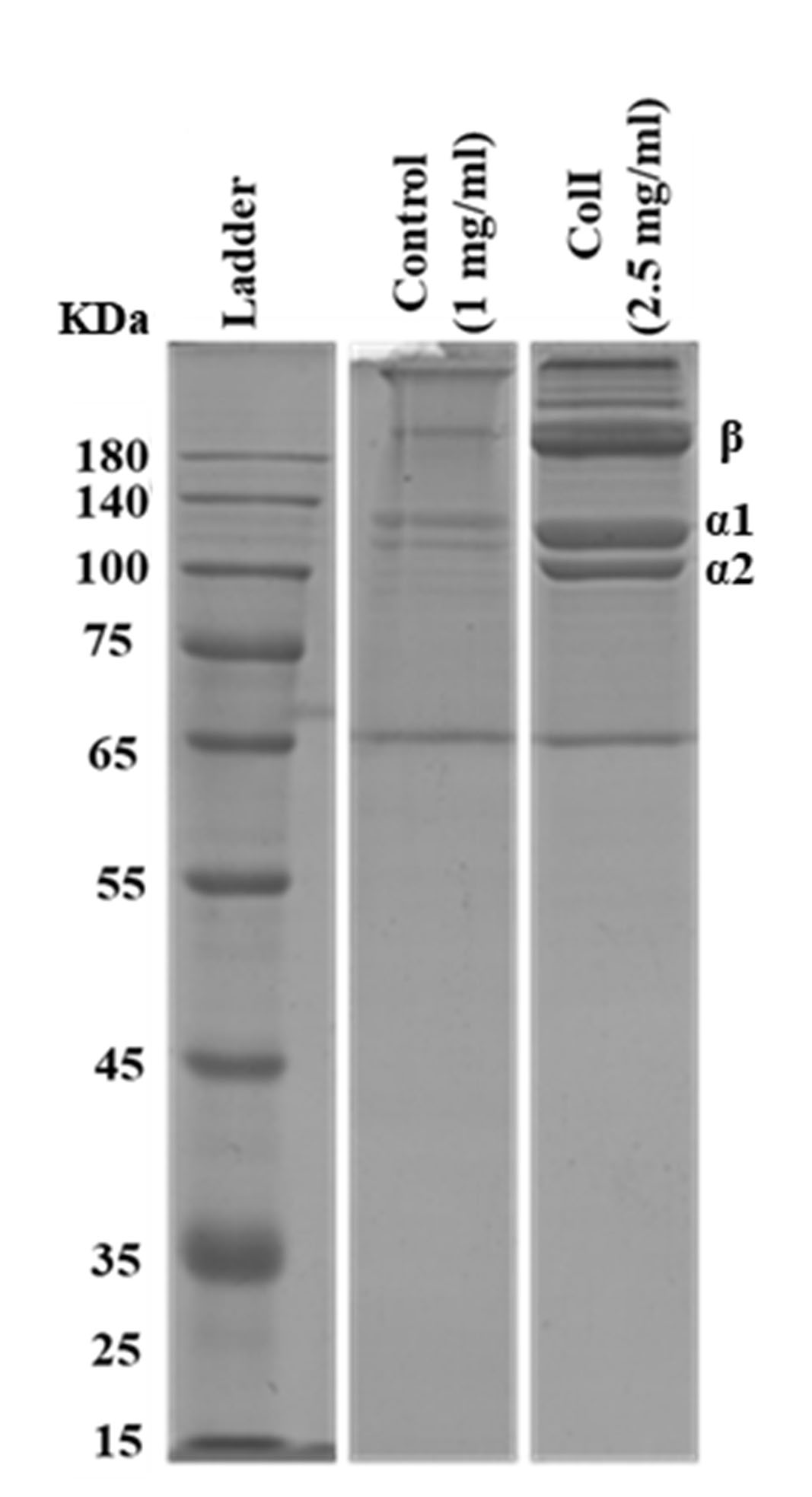
**

**Figure S2.** SDS PAGE results of collagen subunits.

**
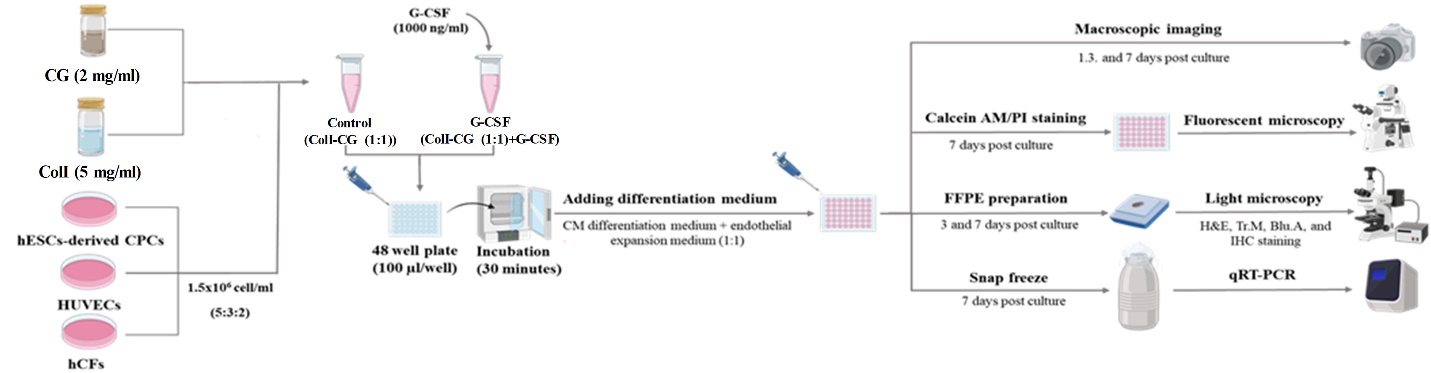
**

**Figure S3.** The schematic outlines of the protocol for cardiac microtissue fabrication under G-CSF-enriched hybrid hydrogel conditions and subsequent evaluations. Cardiac microtissues were generated using CEH-ColI 1:1 hybrid hydrogel alone (Control) and G-CSF-enriched hybrid hydrogel (G-CSF) at a concentration of 1000 ng/ml, achieved through coculturing three cell types: hESCs-CPC, HUVECs, and hCFs, at a density of 1.5×10^6^ cells/ml and a ratio of 5:3:2, respectively.
